# Characterization of virulence factors of *Salmonella* isolated from human stools and street food in urban areas of Burkina Faso

**DOI:** 10.1186/s12866-021-02398-6

**Published:** 2021-12-11

**Authors:** Marguerite E. M. Nikiema, Solange Kakou-ngazoa, Absatou Ky/Ba, Aboubacar Sylla, Evariste Bako, Ameyo Yayra Audrey Addablah, Jean Bienvenue Ouoba, Emmanuel Sampo, Kobo Gnada, Oumarou Zongo, Kuan Abdoulaye Traoré, Adama Sanou, Isidore Juste Ouindgueta Bonkoungou, Rasmata Ouédraogo, Nicolas Barro, Lassana Sangaré

**Affiliations:** 1 Laboratoire de Biologie Moléculaire d’Epidémiologie et de Surveillance des Bactéries et Virus Transmis par les Aliments (LaBESTA). Ecole Doctorale Sciences et Technologies, Université Joseph Ki-Zerbo, 03 BP 7021, Ouagadougou, 03 Burkina Faso; 2 Plateforme de Biologie Moléculaire, Institut Pasteur de Côte d’Ivoire, Abidjan, Côte d’Ivoire; 3 Service de Bactériologie-Virologie, CHU-Yalgado OUEDRAOGO, 03 BP 7022, Ouagadougou, Burkina Faso; 4 Laboratoire de Bactériologie-Virologie, CHU-Bogodogo, Ouagadougou, Burkina Faso; 5 Hôpital Protestant Schiphra, 07 BP 5246, Ouagadougou, 07 Burkina Faso; 6 Centre MURAZ, Bobo-Dioulasso, Burkina Faso; 7 Laboratoire de Biochimie et Immunologie Appliquées (LABIA), Université Joseph Ki-Zerbo, 03 BP 7021, Ouagadougou, 03 Burkina Faso; 8 Université Nazi Boni, 01 BP 1091, Bobo-Dioulasso, 01 Burkina Faso; 9 Laboratoire de Bactériologie-Virologie, CHU-Pédiatrie Charles De Gaulle, 01 BP 1198 BP, Ouagadougou, 01 Burkina Faso

**Keywords:** *Salmonella*, Serotypes, Virulence genes, Gastroenteritis, Sandwiches, Burkina Faso

## Abstract

**Background:**

This study was undertaken to identify and functionally characterize virulence genes from *Salmonella* isolates in street food and stool cultures. From February 2017 to May 2018, clinical and food *Salmonella* strains were isolated in three regions in Burkina Faso. *Salmonella* was serotyped according to the White-Kauffmann-Le Minor method, and polymerase chain reaction (PCR) was used to detec *inv*A, *spvR*, *spvC*, *fimA* and *stn* virulence genes commonly associated with salmonellosis in Sub-Saharan Africa.

**Results:**

A total of 106 *Salmonella* isolates (77 human stools; 14 sandwiches) was analyzed using a serological identification with an O-group test reagent. The presence of *Salmonella* was confirmed in 86% (91/106) of the samples were reactive (OMA-positive/OMB-positive). *Salmonella* serogroup O:4,5 was the most common serogroup detected (40%; 36/91). *Salmonella* Enteritidis and Typhimurium represented 5.5% (5/91) and 3.3% (3/91), respectively and were identified only from clinical isolates. Furthermore, 14 serotypes of *Salmonella* (12/91 human strains and 2/15 sandwich strains) were evocative of Kentucky/Bargny serotype. For the genetic profile, 66% (70/106) of the *Salmonella* had *inv*A and *stn* genes; 77.4% (82/106) had the *fim*A gene. The *spv*R gene was found in 36.8% (39/106) of the isolates while 48.1% (51/106) had the *spv*C gene. Among the identified *Salmonella* Enteritidis and *Salmonella* Typhimurium isolated from stools, the virulence genes detected were *inv*A (3/5) versus (2/3), *fim*A (4/5) versus (3/3), *stn* (3/5) versus (2/3), *spv*R (4/5) versus (2/3) and *spv*C (3/5) versus (2/3), respectively.

**Conclusion:**

This study reports the prevalence of *Salmonella* serotypes and virulence genes in clinical isolates and in street foods. It shows that food could be a significant source of *Salmonella* transmission to humans. Our results could help decision-making by the Burkina Faso health authority in the fight against street food-related diseases, in particular by training restaurateurs in food hygiene.

## Background

*Salmonella* is one of the most problematic foodborne and zoonotic pathogens that threaten general health and well-being [1]. *Salmonella* remains the leading cause of bacterial gastroenteritis and is also one of the most extensively studied and well-characterized bacterial species [2]. In spite of that *Salmonella* continue to be remains an important human pathogen and a serious public health concern worldwide [3]. Salmonellosis, caused by *Salmonella*, manifests mainly as mild diarrhea, also known as food poisoning [4].

In Europe, the number of non-typhoidal salmonellosis (NTS) is estimated at 690 cases per 100,000 inhabitants [5], and in the United States, 17.6 cases per 100,000 inhabitants per year [6]. In 2019, pathogens responsible for foodborne diseases, including *Salmonella enterica* (non-typhoid), caused 230,000 deaths in Africa [7]. In resource-limited countries, particularly in Sub-saharan Africa, NTS is endemic, with high rates in children under 3 years of age and immunocompromised individuals [8, 9]. These diseases have a significant negative economic impact in resource-limited countries amounting to $ 110 billion per year [10].

The pathogenicity of *Salmonella* is mediated by numerous genes comprising *inv*A, *fim*A, *stn*, *spv*R, *spv*C, *spi*C and *pip*D [11]. The invasion gene (*inv*A), located on the pathogenicity island 1 (SPI1), has been widely studied for its ability to promote virulence and also as a biomarker for the detection of *Salmonella* spp. [12]. The *inv*A gene of *Salmonella* is also involved invasion of host epithelial cells [13, 14]. The *Salmonella stn* gene encode for an enterotoxin *stn* and is associated with infection with the serotypes of *Salmonella* Typhi, Typhimurium and Enteritidis [15]. *Salmonella* enterotoxin (*stn*) gene is a clinical important biomarker which is use to differentiate *Salmonella enterica* strains (*stn*+), from in *Salmonella bongori* and other *Enterobacteriaceae* [15]. The fimbriae (*fim*A) are *Salmonella* filamentous surface structures that contribute to colonization of the epithelium cells [16]. *Salmonella* virulence plasmids have been considered as a characteristic of *Salmonella* serotypes implicated in systemic disease. The *Salmonella* virulence plasmid carry several key virulence factors, including the *spv*ABCD system and its *spv*R regulator, which are essential for systemic virulence [17]. These genes are also sufficient to restore systemic virulence in plasmid hardened strains [18]. Although *Salmonella* is a major cause of foodborne illness in developing countries, there is a scarcity of data on street found intake and *Salmonella* related diseases which undermined the real impact of salmonellosis on population health [19]. Other non-typhoidal *Salmonella* (NTS) serovars can also cause systemic infections, also known as invasive NTS (iNTS) disease [19–21]. This is predominantly due to the emergence of invasive clones of Enteritidis [22] and Typhimurium [23] serotypes that have spread throughout Africa. Despite being a serious public health concern, there are very few studies on salmonellosis, associated with food-borne illness, similarly, data on *Salmonella* virulence genes remain limited in Burkina Faso [24]. On the other hand, studies have been reported on the resistance genes of these bacteria [25, 26]. Therefore, there is a need to improve our understanding on the pathogenicity of these bacteria in regards to virulence genes and their impact on human health in Burkina Faso. This work aimed to address this lack of data on salmonellosis through determination of the prevalence of virulence genes of *Salmonella* strains isolated from street food and human stools in Burkina Faso.

## Methods

### Site and period of the study

**Fig. 1:**
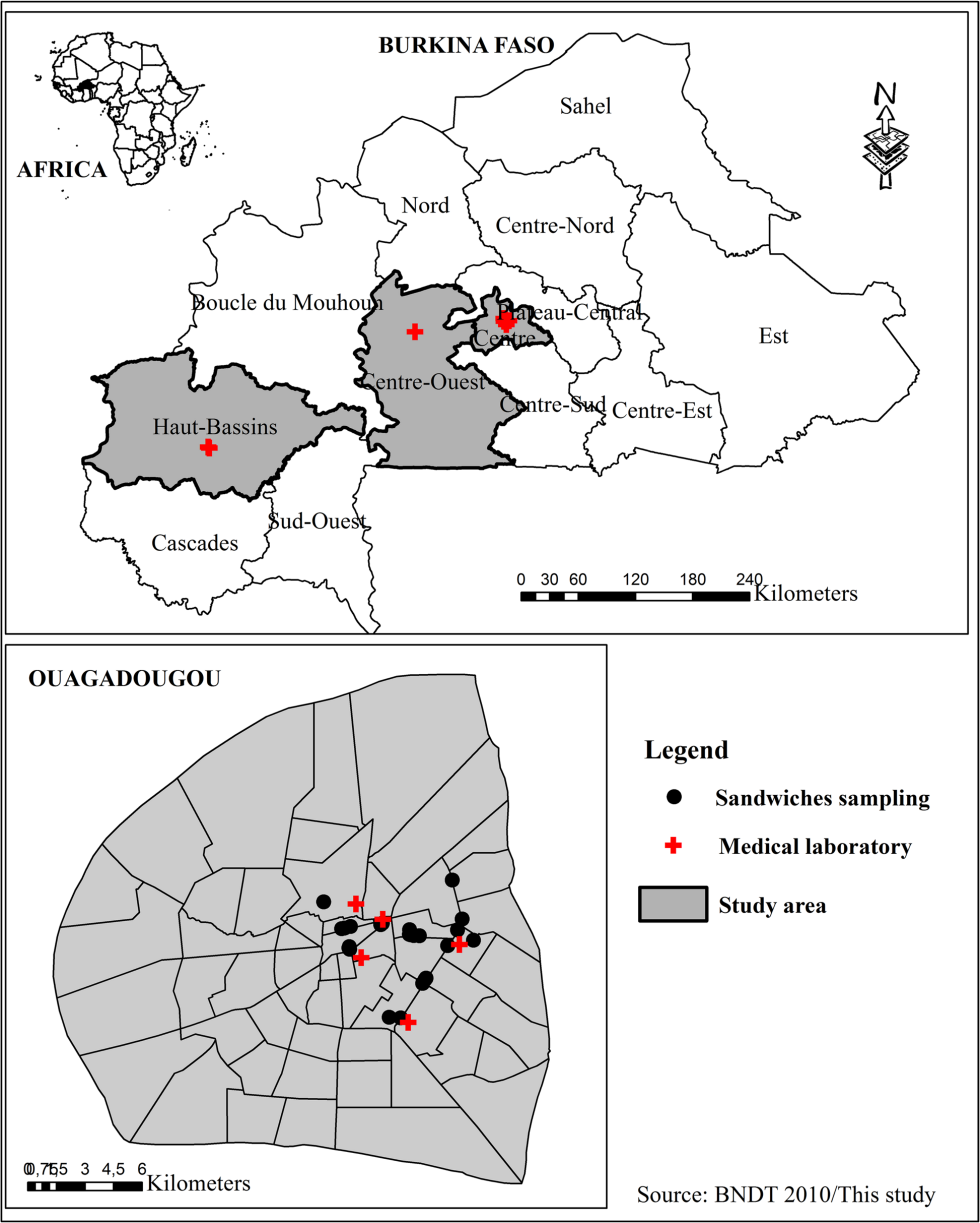
Geographic map of sample collection sites (health facilities and food sites)

Samples were collected from February 2017 to May 2018 in Ouagadougou the capital, and Bobo-Dioulasso and Koudougou located in West and mid-western parts of Burkina Faso (Fig. 1).

### Sample’s collection

Eighty-five (85) samples of ready-to-eat beef sandwiches were purchased in Ouagadougou and transported to the laboratory in a + 4 °C cooler for microbiological analysis. Analysis was carried out within 2 h after sampling. Ninety-one (91) *Salmonella* clinical strains, isolated from diarrheal stools samples from inpatients and out patients were collected from seven health care facilities in urban areas of three regions of Burkina Faso. Central region/Ouagadougou (“Centre Hospitalier Universitaire Yalgado Ouédraogo (CHU-YO)”, “Centre Hospitalier Universitaire Pédiatrique Charles De Gaulle (CHU-PCDG)”, “Centre Hospitalier Universitaire Bogodogo (CHU-B)”, “Hôpital Protestant Schiphra” and “Laboratoire d’analyse médical du Centre”), Hauts-Bassins region/ Bobo-Dioulasso (“Centre MURAZ”) and center-west region/ Koudougou (“Centre Hospitalier Régional de Koudougou”).

Sandwich samples were analyzed at the “Laboratoire de Biologie Moléculaire d’Epidémiologie et de Surveillance des Bactéries et Virus Transmis par les Aliments (LaBESTA, Université Joseph Ki-Zerbo)”.

### Microbiological analyzes

*Salmonella* strains were isolated from eighty-five (85) samples of ready-to-eat beef sandwiches using ISO 6579-1:2017 standard - Horizontal method for *Salmonella* detection of *Salmonella* [27]. The Ninety-one (91) *Salmonella* clinical isolates collected from health facilities of three cities in Burkina Faso (83 in Ouagadougou, 2 in Koudougou and 6 in Bobo-Dioulasso) were submitted to API 20E identification system of Biomérieux France at the CHU-YO laboratory for verify that they are indeed *Salmonella* strains.

#### Pre-enrichment in non-selective broth

Prior to enrichment, 25 g from each sandwich sample were suspended in a sterile flask containing 225 mL of non-selective buffered peptone water (Liofilchem® Srl, Italy) and incubated at 37 °C for 16 to 20 h. This approach helps increase the number of bacterial cells of interest by enabling repair of lesions from damaged cells which regain their resistance to selective agents,

#### Selective enrichment in broth

Following the non-selective pre-enrichment stage, 1 mL and 0.1 mL of each sample suspension were transferred into 10 mL of Muller-Kauffmann Tetrathionate-Novobiocin Broth (MKTTn) (Liofilchem® Srl, Italy) and into 10 mL of Rappaport Vassiliadis Soy broth (Difco laboratories), respectively. Brilliant green at 0.95% was added to the Tetrathionate broth in order to inhibit the growth of Gram-positive bacteria and then incubated for 18 to 20 h at 37 ± 1 °C. The Rappaport Vassiliadis inoculate were incubated for 18 to 20 h at 42 ± 1 °C.

#### Selective isolation

During the third stage, a loopful of culture suspension from each selective media was placed on two different agar plates to identify individual colonies. The media chosen were Xylose Lysine Deoxycholate agar (XLD, HiMedia Laboratories, India) and *Salmonella-Shigella* agar (SS, HiMedia Laboratories, India). Typical *Salmonella* colonies on XLD are colorless, very light, slightly shiny and transparent (colour of the medium) with a dark tinted centre, surrounded by a light red area and yellow edge, but they can also appear as pink to red coloured, with or without a black centre. On the *Salmonella-Shigella* agar, typical *Salmonella* colonies are colourless or very light pink, opaque or semi-transparent, usually with a black centre.

#### Biochemical identification of characteristic colonies

At least five colonies suspicious for *Salmonella* were picked per plate and purified by growth on nutrient agar for 24 h. Then, colonies were sowed onto triple sugar iron agar (Difco laboratories) to observe sugar utilization, MR-VP broth for Voges Proskauer reaction, Christensen agar for urea utilization and peptone water broth for indole production. *S. enterica* serotype Typhimurium strain ATCC 14028 and *S. enterica* serotype Enteritidis strain ATCC 13076 were used as positive controls. Suspected colonies were purified on nutrient agar and then submitted to an API 20E (BioMérieux, Marcy Etoile, France) test for biochemical identification. The main biochemical tests are glucose fermentation, ortho phenyl beta galactosidase negative reaction, urease negative, lysine decarboxylase, negative indole test, H_2_S production, and fermentation of dulcitol [28]. Isolates of *Salmonella* spp. were stored in brain heart broth (BioMérieux, France) supplemented with 30% glycerol, in cryotubes at − 80 °C.

### Serotyping

Serotyping and molecular characterization were performed at the Pasteur Institute in Côte d’Ivoire. All the strains were serotyped according to the White-Kauffmann-Le Minor scheme [29]. We purchased the anti-*Salmonella* agglutinating serums from Bio-Rad (Marnes-la-Coquette, France).

### Molecular characterization of *Salmonella* strains

#### Genomic DNA extraction

The extraction of total genomic DNA was carried out by the phenol/chloroform method [30] using *Salmonella* fresh growths from Luria Bertani (LB) broth. The *Salmonella* strains were grown in LB 1X broth incubated at 37 °C for 24 h. We incubated the mixture of 100 μL of enriched sample added to 300 μL of lysis buffer at 56 °C for 1 h. A total volume of 400 μL of the phenol/chloroform/isoamyl alcohol mixture (25:24:1) was vortexed for 2 min and then centrifuged at 12500 rpm for 2 min. Then the supernatant was transferred in a new 1.5 mL Eppendorf tube and the pellet was discarded. The volume of the transferred supernatant was recorded and a double volume of absolute ethanol (100%) was added to the supernatant. The mixture was incubated for an hour at − 20 °C and then centrifuged at 12500 rpm for 10 min. The supernatant was removed and the visible pellet was retained, washed with 200 μL of 70% of cold ethanol and centrifuged at 12500 rpm for 10 min. Finally, the pellet was dried for 20 min at room temperature and 60 μL of elution buffer was added to the pellet before storage at − 20 °C for PCR.

#### Detection of virulence genes by PCR

*Salmonella* isolates were tested for different virulent genes (*inv*A, *fim*A, *stn*, *spv*R and *spv*C) using PCR with sets of specific primer pairs (Table 1) as described by Chaudhary et al. [31]. The amplification of the genes was carried out according to the method described by Li et al. [12] with minor modifications. Initially, the amplification of the *inv*A gene also served as a specific biomarker for the identification of the genus *Salmonella* [12]. The amplifications of the *inv*A, *spv*C genes were performed following simplex PCR at a different hybridization temperature of 63 °C using a thermocycler an Applied Biosystem GeneAmp PCR System 9700 type and GoTaq® G2 Flexi DNA Polymerase. The reaction mixture, with a final volume of 50 μL, consisted of 1X of 5X Green GoTaq® Flexi Buffer, 2 mM of MgCl2 Solution, 0.2 μM of each primer and PCR Nucleotide Mix, 27.75 μL of sterile distilled water, 1.25 U of GoTaq® G2 Flexi DNA Polymerase (Promega) and 5 μL of DNA extract. Gene amplification was performed as described by Kumar et al. [32] with minor modifications (volume of water and DNA). Then, the PCR conditions for amplification of virulence genes (*inv*A, *spv*C) were as follows: 5 min of initial denaturation at 94 °C, followed by 35 cycles of denaturation at 94 °C for 30 s, hybridization at 63 °C for 30 s, and extension at 72 °C for 30 s, ending with a final extension period of 72 °C for 10 min. Amplifications of the *fim*A, *stn*, *spv*R genes were performed in multiplex with slight variations in volume compared to the previous simplex PCR, using hybridization temperature of 56 °C. With a final volume of 50 μL, the reaction mixture has the same concentrations as described above for the kit GoTaq® G2 Flexi DNA Polymerase.

**Table 1:** The primer pairs used for the characterization of virulence genes from isolates of *Salmonella* [31]

Genes	Primer sequence (5′ → 3′)	TH (°C)	Size (bp)	Reference
*inv*A	F: GTG AAA TTA TCG CCA CGT TCG GGC AA R: TCA TCG CAC CGT CAA AGG AAC C	63	284	[32]
*spvR*	F: CAG GTT CCT TCA GTA TCG CA R: TTT GGC CGG AAA TGG TCA GT	57	310	[33]
*spvC*	F: ACT CCT TGC ACA ACC AAA TGC GGA R: TGT CTT CTG CAT TTC GCC ACC ATC A	63	571	[34]
*fimA*	F: CCT TTC TCC ATC GTC CTG AA R: TGG TGT TAT CTG CCT GAC CA	56	85	[35]
*stn*	F: CTT TGG TCG TAA AAT AAG GCG R: TGC CCA AAG CAG AGA GAT TC	55	260	[36]

### Electrophoresis and band visualization

The amplicons were separated by electrophoresis on a 2% agarose gel containing 8 μg/mL of the intercalant Sybr safe DNA gel Strain (10,000X, Invitrogene, Carlsbad, CA 92008 USA). A volume of 8 μL of each of the PCR products was loaded into each well. Five microliters (5 μL) of a 100 bp DNA Ladder molecular weight marker (Promega, Madison, WI 53704 USA) was used to estimate the size of the amplicons. Electrophoresis was carried out at 110 V for 20 min using the Enduro gel XL electrophoresis system (Labnet, FL, USA). The bands were visualized using the GEL DOC EZ imaging system (Bio-Rad, USA).

## Results

### *Salmonella* strains isolated from food and clinical samples

Fifteen (15) *Salmonella* was isolated from sandwich samples and 91 from clinical samples. In total one hundred and six (106) *Salmonella* strains were involved in the analysis.

### Serotyping of *Salmonella* strains

All 106 *Salmonella* isolates were agglutinated with antisera OMA and OMB. Ninety-one isolates (85.9%) gave a positive result (56 OMA + and 35 OMB +) and the remaining 15 isolates were negative to OMA and OMB. *Salmonella* of the antigenic groups OMA+/O:4.5 [39.6% (36/91)] and the antigenic group OMB+/O:6,7,8 [25.3% (23/91)] were the most frequent. Three (3) *Salmonella* Typhimurium serotypes (OMA+/O:4.5; HMB+/H:i H2:gm) and five (5) *Salmonella* Enteritidis serotypes (OMA+/O:9; HMB+/H1:gm H2:-) were found, only among clinical isolates. In addition, 14 strains of antigenic formulas (OMB+/O:6,7,8; HMA+/H:i) evocative of the Kentucky/Bargny serotype were isolated (2 from sandwiches and 12 from clinical isolates). These 14 *Salmonella* isolates were called suspected Kentucky serotypes because the serotyping was incomplete. All reported strains of *Salmonella* could not be completely serotyped by available sera as showed in Table 2.

**Table 2:** Prevalence of *Salmonella* serotypes and virulence genes

Origin strains	***Salmonella*** serotypes (serogroups)	Number	Genetic markers				
			***inv***A	***fim***A	***stn***	***spv***R	***spv***C
Clinical	Enteritidis [D (9)]	5	3	4	3	4	3
	Typhimurium [B (4)]	3	2	3	2	2	2
	Kentucky/Bargny^a^[C (8)]	12	8	8	8	3	6
	*Salmonella* spp.	71	56	63	55	26	39
	Total	91	61 (67%)	70 (76.9%)	60 (65.9%)	32 (35.2%)	44 (48.4%)
Food	Kentucky/Bargny^a^[C (8)]	2	1	2	1	1	1
	*Salmonella* spp.	13	9	12	10	7	7
	Total	15	9 (60%)	12 (80%)	10 (66.7%)	7 (46.7%)	7 (46.7%)
Total (*n* = 106)			70 (66%)	82 (77.4%)	70 (66%)	39 (36.8%)	51 (48.1%)

^a^antigenic formula (OMB+/O:6,7,8; HMA H:i) suggestive of *Salmonella* Kentucky/Bargny

### Molecular characterization of *Salmonella* strains

#### Molecular identification of *Salmonella* strains

The molecular identification of *Salmonella* strain showed that among the 106 *Salmonella* isolates, 70 (66%) carried *inv*A genes, from which 61 (87.1%) were clinical isolates, and 9 (12.9%) were isolated from sandwich samples (Table 2).

#### Molecular detection of *Salmonella* virulence genes

The most prevalent virulence genes were *fim*A, 82/106 (77.4%) followed by *stn* and *inv*A 70/106 (66%), *spv*C 51/106 (48.1%) and *spv*R 39/106 (36.8%).

The distribution of amplified genes according to strain origin (Table 2), showed that 67% (61/91) of clinical strains carried *inv*A gene, 76.9% (70/91) *fim*A gene, 65.9% (60/91) *stn* gene, 35.2% (32/91) *spv*R gene and 48.4% (44/91) *spv*C gene.

*Salmonella* isolated from food (sandwich) carried *fim*A genes at 80% (12/15), *stn* genes at 66.7% (10/15), *inv*A genes at 60% (9/15), *spv*R and *spv*C genes at 46.7% (7/15).

The distribution of virulence genes according to the serotypes from clinical isolates showed that 80% (4/5) of *Salmonella* Enteritidis strains carried *fim*A and *spv*R genes, and 60% (3/5) carried *inv*A, *stn* and *spv*C. 100% (3/3) of *Salmonella* Typhimurium carried *fim*A and *spvC* genes, and 66.7% (2/3) carried *inv*A, *stn*, *fim*A and *spv*R genes.

The majority of the 106 *Salmonella* isolates harbored at least one of the five genes associated with virulence (Table 3, Table 4). According to the presence of virulence genes, we classified the 106 isolates in eleven (11) different profiles (P) from clinical isolates and named as follows: P1, P2, P3, P10 to P11. The foodborne *Salmonella* had five (5) genetic profiles (P1, P3, P10, P7 and P11). For all isolates combined (food and human); profile P1 (positive for all five genes tested) was the most prevalent 38 (35.8%), follow by P2 (*spv*R absent), 12 (11.3%), profile P3 (*spv*R and *spv*C absent), 16 (15.1%), P10 (unique presence of *fim*A), 10 (9.4%) and P11 (negative for all five genes tested), 22 (20.8%).

**Table 3:** Virulence genes and genetic profile of *Salmonella* spp. isolates from food in Burkina Faso

***Salmonella*** Strain code	Year	City	Source	Serotypes	Antigenic formule positive	Identified genes	Genetic profile
93	2017	Ouagadougou	Food	ICS	OMA+ O:3,1,15; HMA	*inv*A, *fim*A, *stn*, *spv*R, *spv*C	P1
94	2017	Ouagadougou	Food	ICS	OMA+ O:4,5; HMB	*inv*A, *fim*A, *stn*, *spv*R, *spv*C	P1
96	2017	Ouagadougou	Food	ICS	OMA+ O:4,5; O:1,2; HMB Hgm	*inv*A, *fim*A, *stn*, *spv*R, *spv*C	P1
97	2017	Ouagadougou	Food	ICS	OMB+ O:6,7,8	*inv*A, *fim*A, *stn*, *spv*R, *spv*C	P1
98	2017	Ouagadougou	Food	ICS	OMA+ O:4,5; HMA	*inv*A, *fim*A, *stn*, *spv*R, *spv*C	P1
100	2017	Ouagadougou	Food	ICS	OMB+	*inv*A, *fim*A, *stn*, *spv*R, *spv*C	P1
104	2017	Ouagadougou	Food	ICS	OMB+ O:6,7,8; HMA Hi*^a^	*inv*A, *fim*A, *stn*, *spv*R, *spv*C	P1
92	2017	Ouagadougou	Food	ICS	OMA+ O:4,5; HMA	*inv*A, *fim*A, *stn*	P3
95	2017	Ouagadougou	Food	ICS	OMA+ O:4,5; O:1,2; HMB	*inv*A, *fim*A, *stn*	P3
103	2017	Ouagadougou	Food	ICS	OMA+ O:4,5; HMB Hg^b^	*fim*A, *stn*	P7
102	2017	Ouagadougou	Food	ICS	OMA+ O:4,5; HMB Hg^c^	*fim*A	P10
105	2017	Ouagadougou	Food	ICS	OMB+ O:6,7,8; HMA Hi*	*fim*A	P10
99	2017	Ouagadougou	Food	ICS	OMA+ O:4,5 O1,2	…	P11
101	2017	Ouagadougou	Food	ICS	OMA-OMB-	…	P11
106	2017	Ouagadougou	Food	ICS	OMA+ O:3,1015; HMB	…	P11

*ICS* Incomplete serotyping, *OMA* Antiserum O mixture of group A, *OMB* Antiserum O mixture of group B, *HMA* Antiserum H mixture of group A, *HMB* Antiserum H mixture of group B; *antigenic formula (OMB+/O:6,7,8; HMA H:i) suggestive of *Salmonella* Kentucky/Bargny; P1: positive for all five genes tested; P3: *spv*R and *spv*C absent; P7: unique presence of *fimA* and *stn*; P10: unique presence of *inv*A, P11: negative for all five genes test; ^a, b, c^overlapping serotypes/genetic profile

**Table 4:** Virulence genes and genetic profile of *Salmonella* spp. isolates from human stool in Burkina Faso

***Salmonella*** Strain code	Ye ar	City	Source	Serotypes	Antigenic formule positive	Identified genes	Genetic profile
44	2018	Ouagadougou	Human	ICS	OMB+ O:6,7,8	*inv*A, *fim*A, *stn*, *spv*R, *spv*C	P1
45	2018	Ouagadougou	Human	ICS	OMA-OMB-	*inv*A, *fim*A, *stn*, *spv*R, *spv*C	P1
46	2018	Ouagadougou	Human	ICS	OMA+ O:4,5, HMB Hgm	*inv*A, *fim*A, *stn*, *spv*R, *spv*C	P1
48	2018	Ouagadougou	Human	ICS	OMB+ O:6,7,8; HMA Hi	*inv*A, *fim*A, *stn*, *spv*R, *spv*C	P1
50	2018	Ouagadougou	Human	ICS	OMB+ O:6,7,8; HMB Hg	*inv*A, *fim*A, *stn*, *spv*R, *spv*C	P1
51	2018	Ouagadougou	Human	ICS	OMA+ O:4,5; HMB Hg	*inv*A, *fim*A, *stn*, *spv*R, *spv*C	P1
53	2018	Ouagadougou	Human	Typhimurium	OMA+ O:4,5; HMA Hi; H1H2	*inv*A, *fim*A, *stn*, *spv*R, *spv*C	P1
59	2017	Ouagadougou	Human	ICS	OMA-OMB-	*inv*A, *fim*A, *stn*, *spv*R, *spv*C	P1
68	2017	Ouagadougou	Human	ICS	OMA+ O:4,5; HMB Hg	*inv*A, *fim*A, *stn*, *spv*R, *spv*C	P1
71	2018	Ouagadougou	Human	ICS	OMA-OMB-	*inv*A, *fim*A, *stn*, *spv*R, *spv*C	P1
74	2017	Koudougou	Human	Enteritidis	OMA+ O:9; HMB Hgm	*inv*A, *fim*A, *stn*, *spv*R, *spv*C	P1
75	2017	Koudougou	Human	Enteritidis	OMA+ O:9; HMB Hgm	*inv*A, *fim*A, *stn*, *spv*R, *spv*C	P1
85	2017	Ouagadougou	Human	ICS	OMA+ O:4,5, HMB Hgm	*inv*A, *fim*A, *stn*, *spv*R, *spv*C	P1
86	2017	Ouagadougou	Human	ICS	OMA-OMB-	*inv*A, *fim*A, *stn*, *spv*R, *spv*C	P1
3	2017	Bobo-Dioulasso	Human	Enteritidis	OMA+ O:9; HMB Hgm	*inv*A, *fim*A, *stn*, *spv*R, *spv*C	P1
17	2017	Ouagadougou	Human	ICS	OMA+ O:9	*inv*A, *fim*A, *stn*, *spv*R, *spv*C	P1
18	2018	Ouagadougou	Human	ICS	OMB+ O:6,7,8	*inv*A, *fim*A, *stn*, *spv*R, *spv*C	P1
19	2018	Ouagadougou	Human	ICS	OMA-OMB-	*inv*A, *fim*A, *stn*, *spv*R, *spv*C	P1
27	2018	Ouagadougou	Human	ICS	OMA+ O:4,5; HMB, Hg	*inv*A, *fim*A, *stn*, *spv*R, *spv*C	P1
28	2018	Ouagadougou	Human	ICS	OMA-OMB-	*inv*A, *fim*A, *stn*, *spv*R, *spv*C	P1
29	2018	Ouagadougou	Human	Typhimurium	OMA+ O:4,5; HMA Hi; H1H2	*inv*A, *fim*A, *stn*, *spv*R, *spv*C	P1
30	2017	Ouagadougou	Human	ICS	OMA+ O:3,10,15; HMA Hi	*inv*A, *fim*A, *stn*, *spv*R, *spv*C	P1
32	2017	Ouagadougou	Human	ICS	OMA+ O:4,5, HMB	*inv*A, *fim*A, *stn*, *spv*R, *spv*C	P1
34	2017	Ouagadougou	Human	ICS	OMA+ O:9	*inv*A, *fim*A, *stn*, *spv*R, *spv*C	P1
35	2017	Ouagadougou	Human	ICS	OMB+ O:6,7,8; HMA Hi*	*inv*A, *fim*A, *stn*, *spv*R, *spv*C	P1
36	2017	Ouagadougou	Human	ICS	OMB+ O:6,7,8; HMA Hi*	*inv*A, *fim*A, *stn*, *spv*R, *spv*C	P1
37	2017	Ouagadougou	Human	ICS	OMA-OMB-	*inv*A, *fim*A, *stn*, *spv*R, *spv*C	P1
38	2017	Ouagadougou	Human	ICS	OMA-OMB-	*inv*A, *fim*A, *stn*, *spv*R, *spv*C	P1
39	2017	Ouagadougou	Human	ICS	OMA+ O:9	*inv*A, *fim*A, *stn*, *spv*R, *spv*C	P1
40	2017	Ouagadougou	Human	ICS	OMA+ O:4,5, HMA	*inv*A, *fim*A, *stn*, *spv*R, *spv*C	P1
43	2017	Ouagadougou	Human	ICS	OMA+ O:4,5; HMB	*inv*A, *fim*A, *stn*, *spv*R, *spv*C	P1
1	2017	Bobo-Dioulasso	Human	ICS	OMA+ O:4,5; HMB Hg	*inv*A, *fim*A, *stn*, *spv*C	P2
4	2017	Bobo-Dioulasso	Human	ICS	OMA+ O:4,5; HMB,Hgm	*inv*A, *fim*A, *stn*, *spv*C	P2
20	2018	Ouagadougou	Human	ICS	OMB+	*inv*A, *fim*A, *stn*, *spv*C	P2
21	2018	Ouagadougou	Human	ICS	OMB+	*inv*A, *fim*A, *stn*, *spv*C	P2
22	2018	Ouagadougou	Human	ICS	OMA+ O:4,5, HMB	*inv*A, *fim*A, *stn*, *spv*C	P2
23	2018	Ouagadougou	Human	ICS	OMA-OMB-	*inv*A, *fim*A, *stn*, *spv*C	P2
24	2018	Ouagadougou	Human	ICS	OMA-OMB-	*inv*A, *fim*A, *stn*, *spv*C	P2
25	2018	Ouagadougou	Human	ICS	OMA-OMB-	*inv*A, *fim*A, *stn*, *spv*C	P2
26	2018	Ouagadougou	Human	ICS	OMB+ O:6,7,8	*inv*A, *fim*A, *stn*, *spv*C	P2
80	2017	Ouagadougou	Human	ICS	OMB+ O:6,7,8; HMA Hi*	*inv*A, *fim*A, *stn*, *spv*C	P2
81	2017	Ouagadougou	Human	ICS	OMB+ O:6,7,8; HMA Hi*	*inv*A, *fim*A, *stn*, *spv*C	P2
84	2017	Ouagadougou	Human	ICS	OMB+ O:6,7,8; HMA Hi*	*inv*A, *fim*A, *stn*, *spv*C	P2
2	2017	Bobo-Dioulasso	Human	ICS	OMB+ O:6,7,8	*inv*A, *fim*A, *stn*	P3
5	2017	Bobo-Dioulasso	Human	ICS	OMB+	*inv*A, *fim*A, *stn*	P3
6	2017	Bobo-Dioulasso	Human	ICS	OMB+ O:6,7,8; HMA Hi*	*inv*A, *fim*A, *stn*	P3
7	2017	Ouagadougou	Human	ICS	OMA+ O:4,5; HMB,Hgm	*inv*A, *fim*A, *stn*	P3
8	2017	Ouagadougou	Human	ICS	OMB+ O:6,7,8; HMA	*inv*A, *fim*A, *stn*	P3
9	2017	Ouagadougou	Human	ICS	OMB+ O:6,7,8; HMA, Hi*	*inv*A, *fim*A, *stn*	P3
10	2017	Ouagadougou	Human	ICS	OMA+ O:4,5; HMB,H1	*inv*A, *fim*A, *stn*	P3
11	2017	Ouagadougou	Human	ICS	OMB+	*inv*A, *fim*A, *stn*	P3
14	2017	Ouagadougou	Human	ICS	OMA+	*inv*A, *fim*A, *stn*	P3
15	2017	Ouagadougou	Human	ICS	OMA+	*inv*A, *fim*A, *stn*	P3
16	2017	Ouagadougou	Human	ICS	OMA+ O:9	*inv*A, *fim*A, *stn*	P3
54	2018	Ouagadougou	Human	ICS	OMB+	*inv*A, *fim*A, *stn*	P3
55	2018	Ouagadougou	Human	ICS	OMA+ O:4,5; HMB Hg	*inv*A, *fim*A, *stn*	P3
73	2018	Ouagadougou	Human	ICS	OMA+ O:4,5; HMB Hg	*inv*A, *fim*A, *stn*	P3
72	2017	Ouagadougou	Human	ICS	OMB+ O:6,7,8	*fim*A, *stn*, *spv*C	P4
13	2017	Ouagadougou	Human	ICS	OMA+ O:4,5, HMB	*inv*A, *fim*A	P5
31	2017	Ouagadougou	Human	ICS	OMA-OMB-	invA, fimA	P5
33	2017	Ouagadougou	Human	ICS	OMA+ O:3,10,15	*inv*A, *stn*	P6
58	2017	Ouagadougou	Human	ICS	OMA+ O:4,5, HMB Hgm^b^	*fim*A, *stn*	P7
57	2017	Ouagadougou	Human	Enteritidis	OMA+ O:9; HMB Hgm	*fim*A, *spv*R	P8
12	2017	Ouagadougou	Human	ICS	OMA+	*inv*A	P9
42	2017	Ouagadougou	Human	ICS	OMA+ O:3,10,15	*fim*A	P10
56	2017	Ouagadougou	Human	Typhimurium	OMA+ O:4,5; HMA Hi; H1H2	*fim*A	P10
60	2017	Ouagadougou	Human	ICS	OMA+ O:4,5; HMB Hg	*fim*A	P10
62	2017	Ouagadougou	Human	ICS	OMA+ O:4,5; HMB Hg	*fim*A	P10
63	2017	Ouagadougou	Human	ICS	OMA+ O:4,5, HMB Hgm^c^	*fim*A	P10
65	2017	Ouagadougou	Human	ICS	OMA+ O:4,5; HMA Hi	*fim*A	P10
66	2017	Ouagadougou	Human	ICS	OMB+	*fim*A	P10
67	2017	Ouagadougou	Human	ICS	OMA+ O:4,5, HMB Hgm	*fim*A	P10
41	2017	Ouagadougou	Human	ICS	OMA+ O:4,5, HMB Hgm	…	P11
47	2018	Ouagadougou	Human	ICS	OMA-OMB-	…	P11
49	2018	Ouagadougou	Human	ICS	OMA+ O:3,10,15	…	P11
52	2018	Ouagadougou	Human	ICS	OMA + O:3,10,15	…	P11
61	2017	Ouagadougou	Human	ICS	OMA+ O:4,5; HMB	…	P11
64	2017	Ouagadougou	Human	ICS	OMA-OMB-	…	P11
69	2017	Ouagadougou	Human	ICS	OMB+ O:6,7,8; HMB Hgm	…	P11
70	2017	Ouagadougou	Human	ICS	OMB+ O:6,7,8; HMA Hi*	…	P11
76	2017	Ouagadougou	Human	ICS	OMB+ O:6,7,8; HMA Hi*	…	P11
77	2017	Ouagadougou	Human	ICS	OMB+ O:6,7,8; HMA Hi*	…	P11
78	2017	Ouagadougou	Human	ICS	OMB+	…	P11
79	2017	Ouagadougou	Human	ICS	OMB+ O:6,7,8; HMA Hi*	…	P11
82	2017	Ouagadougou	Human	ICS	OMA+ O:4,5	…	P11
83	2017	Ouagadougou	Human	ICS	OMA+ O:4,5	…	P11
87	2017	Ouagadougou	Human	ICS	OMB+	…	P11
88	2017	Ouagadougou	Human	ICS	OMB+ O:6,7,8	…	P11
89	2017	Ouagadougou	Human	ICS	OMB+	…	P11
90	2017	Ouagadougou	Human	Enteritidis	OMA+ O:9; HMB Hgm	…	P11
91	2017	Ouagadougou	Human	ICS	OMB+	…	P11

*ICS* Incomplete serotyping, *OMA* Antiserum O mixture of group A, *OMB* Antiserum O mixture of group B, *HMA* Antiserum H mixture of group A, *HMB* Antiserum H mixture of group B; *antigenic formula (OMB+/O:6,7,8; HMA H:i) suggestive of *Salmonella* Kentucky/Bargny; P1: positive for all five genes tested; P2: *spv*R absent; P3: *spv*R and *spv*C absent; P9: unique presence of *fimA*; P10: unique presence of *inv*A, P11: negative for all five genes test; ^a, b, c^:overlapping serotypes/genetic profile

## Discussion

The present study investigated the frequency of serotypes of *Salmonella*, the prevalence and genetic characteristics of *Salmonella* virulence genes from human diarrheal stools and street-vended sandwiches in Burkina Faso. This is the first study that reports the distribution of *Salmonella* virulence factors isolated from ready-to-eat sandwiches sold in the street in Burkina Faso. Analyses showed that only 15 (17.7%) out of 85 samples from street-vended sandwiches in Ouagadougou were positive for *Salmonella*. Our result is different from those found by other researchers. Hassanin et al. [37] isolated *Salmonella* in 31.1% of shawarmas samples in Egypt, while Abd-El-Malek et al. [38] isolated 7% in kibdas. In Chad, Djibrine et al. [39] did not isolate any *Salmonella* from beef minced sandwiches vended in streets. These variation rates might be linked to the diversity of cooking process in these different countries.

Serotyping revealed the presence of *Salmonella* Enteritidis (5/91), *Salmonella* Typhimurium (3/91), and *Salmonella* Kentucky/Bargny (12/91) serotypes (Table 2). The remaining isolates that have been not identified completely were categorized as *Salmonella* spp. (71/91). *Salmonella* Typhimurium and *Salmonella* Enteritidis serotypes were from clinical strains. Indeed, *Salmonella* Typhimurium and *Salmonella* Enteritidis, are the most serotypes involved in human infections and frequently isolated from farm animals [40]. In Sub-saharan Africa, non-typhoid salmonellosis is endemic and the serogroups B (4), D (9) and C (8) have been identified from *Salmonella* isolated in this study (Table 2). An earlier study argued that more than 2500 serovars of *Salmonella enterica* were identified by using the White-Kauffmann-Le Minor scheme; about 20 serovars were found primarily in antigen groups B, C, D and E [41]. The serogroups B, D and C are the main causes of human infections, including gastroenteritis and bacteremia [42]. Also, they are widely distributed among farm animals and enter the food chain [40]. Our study reported suspected Kentucky serotypes. This ubiquitous serotype has been closely linked to poultry since 1937 and is now spread in several African countries [43]. In the current decade in West Africa, the finding of the expansion of *Salmonella* Kentucky in the poultry sector has also been reported by Igomu et al. [44] and Kagambèga et al. [45].

The virulence of *Salmonella* is linked to a combination of chromosomal and plasmid factors; the *inv*A gene serves also as a specific biomarker for the identification of the genus *Salmonella* [12]. Elsewhere, published papers [31, 46–48] reveal that *inv*A gene has already been detected in 100% of *Salmonella* strains. However, in our study we found a lower rate (66%) to 100%. Other authors like Mthembu et al. [49] reported lower rates of (54.4%; 106/195) and Somda et al. [24] reported the presence of the *inv*A gene in 91% (52/57) of nontyphoidal *Salmonella* isolates from human diarrhea, environment and lettuce samples in Burkina Faso. In our study, 34% of *Salmonella* isolates do not have the *inv*A gene and therefore would be unable to induce host cell invasion. Then, *Salmonella* may be in a virulent (*inv*A) or non-virulent state [50]. In addition, asymptomatic animals’ carrying these virulent or non-virulent strains could be potential sources of their transmission to humans via the food chain, promiscuity between human and animals, and the poor management of animal effluents [49, 50]. In clinical isolates and in food (sandwich) isolates, the *inv*A, *fim*A and *stn* genes found have approximately high percentages and the presence of one would predict the presence of the other. Several authors previously revealed the constant presence of *invA*, *fimA* and *stn* gene in all *Salmonella* isolates analyzed in their study [31, 47, 48]. As observed by Foley et al. [11, 49], the difference in frequency of the virulence genes observed in our study could be related to the topology of the gene in *Salmonella*; despite their different locations, they remain responsible for virulence in salmonellosis. The virulence plasmid gene *spv*R was present in 36.8% of strains, giving them the ability to cause systemic infections. This frequency of *spv*R is significantly higher than those reported by several authors [24, 31, 48]. The *spv*C gene was detected in 48.1% of all the isolates (106) tested, 44/106 were clinical isolates and 7/106 came from sandwiches (Table 2). Krzyzanowski et al. [51] found out a low rate of *Salmonella* strains with the *spv*C gene, suggesting its particularity in the virulence of *Salmonella*. Amini et al. [52] found out the presence of *spv*C in *Salmonella* strains isolated from humans and cattle and reported 100 and 90%, respectively.

Eight (08) *Salmonella* spp. isolated from the sandwiches indicated a simultaneous presence of *spv*C and *spv*R. Three of the *Salmonella* serotype Enteritidis and two of the *Salmonella* serotype Typhimurium identified from clinical isolates also harbored these two genes (*spv*C and *spv*R) (Table 2). Derakhshandeh et al. [53] also found out two human serotypes of *Salmonella* Enteritidis indicating a simultaneous presence of *spv*C and *spv*R. However, Chaudhary et al. [31] reported the complete absence of *spv*C gene in all their analyzed isolates. The *spv*R locus is strongly associated with strains that cause non-typhoid bacteremia, but are not present in typhoid strains [54]. However, in Senegal, none of *Salmonella* serotype Keurmassar investigated (human and poultry origin) harbored a virulence plasmid [46]. This gene is not commonly found in the genome of *Salmonella*, but is of paramount importance when present. In addition, most *Salmonella* Typhimurium strains contain a self-transmissible virulence plasmid (pSLT) such as the *spv* operon [18]. *Salmonella’s* genetic variations could be derived from transfer of this organism between human-origin and animal/food-origin strains [55]. Whether this can transfer virulence plasmid from animal-origin strains to human-origin strains or vice versa remains to be investigated. These genes (*spv*R and *spv*C) are carried by mobile genetic elements that are lost over the time: their distribution, which appears low, does not reflect the clinical reality [56]. Despite our study’s antigenic similarities among the *Salmonella* isolates, the genetic profile was different for all strains. Although all serotypes of *Salmonella* can be considered potentially pathogenic, there have some differences in their virulence [57]. *Salmonella* isolated from sandwiches share same types of virulence genes with clinical isolates. Six *Salmonella* (food and human) isolates overlapped (^a, b,c^) due to their partial antigenic formulas, genetic profiles and locality (Table 3, Table 4). We also found that these overlaps are in city of Ouagadougou, which may justify because food samples were collected in Ouagadougou only and in the vicinity of medical centers (Fig. 1). We could be led to verify the fact that clinical *Salmonella*’s derive from meal contaminations and vice versa. It was possible to characterize the isolates according to different genetic profiles [47]. The P1 profile (positive for all five genes tested) had the highest frequency necessary for a very successful infection, demonstrating that these genes are widely distributed in *Salmonella* population (Table 3, Table 4). Detection of several genetic profiles may suggest gene acquisitions or deletions in different clones, which could favor different levels of adaptation of strains to the host [58]. Then, genetic variations in *Salmonella* could be derived from the transfer of virulence plasmid from animal-origin strains to human-origin strains or vice versa, which remains to be investigated [55]. The P11 profile indicates that strains do not harbor any of the five virulence genes. Our study reported the P11 profile in 20.75% (22/106) of the *Salmonella* (Table 3, Table 4), even though these strains were confirmed by serotyping. Theoretically, in clinical *Salmonella*, the *inv*A and *stn* genes should be present. However, the acquisition of the lactose operon by *Salmonella* reduces its virulence potential [59]. The hypothesis would be that these isolates have lost their virulence genes during their evolution, or are avirulent and the low sensitivity of the PCR.

## Conclusion

This study highlighted the most serotypes frequently involved salmonellosis in Burkina Faso. *Salmonella* Enteritidis and Typhimurim were mainly isolated in human stool. Additional analysis is needed to confirm the plausible presence of the Kentucky/Bargny serotype among the food and clinical isolates. The presence of *Salmonella* virulence genes was equally important in food and clinical isolates. The presence of virulence genes among isolates from sandwich samples alerted on the potential risk of contamination of the population and probably a possible community health crisis. In addition, the results of this study support that there is genetic differentiation between isolates of the same serotype in the distribution of virulent genes. This provides a basis for the criteria for determining possible variations in the virulence of different strains in vivo, as well as further studies in full serotyping and phylogenetic analysis. These results could enable Burkina Faso’s health authority to better orient their programs to fight diseases associated with street food, notably through the training of restaurateurs in food hygiene.

## Data Availability

The datasets used and/or analyzed during the current study available from the corresponding author on reasonable request.

## Abbreviations

NTS: Non-typhoidal *Salmonella*
*stn*: *Salmonella* enterotoxin
XLD: Xylose Lysine Deoxycholate
SS: *Salmonella-Shigella*
MR-VP Broth: Methyl-red Voges-Proskauer Broth
LB Broth: Luria Bertani Broth
PCR: Polymerase Chain Reaction
ICS: Incomplete serotyping
OMA: Antiserum O mixture of group A
OMB: Antiserum O mixture of group B
HMA: Antiserum H mixture of group A
HMB: Antiserum H mixture of group B
pSLT: self-transmissible virulence plasmid
*spv*: *Salmonella* plasmid virulence
